# Assistive technology unmet need and barriers to access in Sri Lanka: a mixed-methods study (2024)

**DOI:** 10.1016/j.lansea.2026.100807

**Published:** 2026-06-24

**Authors:** Dilisha Patel, Jamie Danemayer, Pavithra Jayawardena, Hasintha Wijesekara, Nadeesh de Silva, Maria Kett, Victoria Austin

**Affiliations:** a Global Disability Innovation Hub, Department of Computer Science, University College London, London, UK; b Department of International Relations, University of Colombo, Colombo, Sri Lanka; c Department of Natural Resources, Sabaragamura University of Sri Lanka, Balangoda, Sri Lanka; d Department of Legal Studies, Open University of Sri Lanka, Colombo, Sri Lanka; e Department of Epidemiology and Public Health, University College London, London, UK

**Keywords:** Assistive technology, Disability, Ageing, Mixed-methods

## Abstract

**Background:**

Assistive technology (AT) access supports functional abilities and is considered a crucial component in aged care planning. To address the research gap with respect to AT use, unmet need, and access barriers in Sri Lanka, this study was undertaken using a mixed methods approach.

**Methods:**

Using WHO tools, the government of Sri Lanka undertook a country capacity Assistive Technology Assessment (ATA-C) in combination with the rapid Assistive Technology Assessment (rATA) survey in 2024. The rATA consisted of a nationally representative household survey including all household members greater than 2 years of age to estimate AT use, need, and unmet need, while the ATA-C guided qualitative data collection through 26 key informant interviews and six focus group discussions. Logistic regression tested the association of demographic variables with AT access indicators in weighted survey data, producing odds ratios and predicted prevalences. Qualitative data were analysed using reflexive thematic analysis, to understand access barriers. An adapted Delphi method was adopted to build consensus on the recommendations during the final stakeholder workshop.

**Findings:**

An estimated 35.2% (95% CI 33.9–36.6) of the study population experienced functional difficulty; 18.1% (16.9–19.3) reported using AT, while 16.4.% (15.4–17.6) reported unmet AT needs, with higher unmet need among older adults, females, and rural and estate residents. Key barriers included cost, information/awareness gaps, sociocultural/environmental factors, and provision/coordination challenges, which differentially impacted groups within the population.

**Interpretation:**

Age, sex, and setting were associated with AT access disparities. Their interaction and relationship to systemic barriers must be considered alongside population ageing to create equitable AT policies.

**Funding:**

Asian Development Bank.

**Research in context**

**Evidence before this study**

Assistive technology (AT) includes products such as wheelchairs, hearing aids, and digital tools, alongside the services required for their provision, and is essential for enabling functional ability, independence, and social and economic participation. Globally, unmet need for AT remains high and is shaped by demographic, socioeconomic, and health system factors. Sri Lanka is experiencing rapid population ageing, increasing demand for AT, yet evidence on need, use, unmet need, and access barriers is extremely limited. We searched PubMed on March 24, 2026, for full-length original articles with South Asian country names and terms related to assistive technology in their title/abstract. We identified seven studies using population-based data, with none conducted in Sri Lanka or using a mixed-methods approach. Previous studies show variation in AT indicators by demographic factors and identify common barriers, including cost and awareness, but are typically based on targeted samples and lack nationally representative sample or policy-oriented analyses, limiting understanding of how barriers arise and can be addressed.

**Added value of this study**

This study employs a mixed-methods approach to estimate AT need, use, and unmet need; and understand access barriers in Sri Lanka, utilising quantitative data from 6100 survey participants along with qualitative data from 26 key informant interviews and six focus group discussions. Among survey participants, 35.2% (95% CI 33.9–36.6) reported functional difficulty, 18.1% (16.9–19.3) reported using AT, and 16.4% (15.4–17.6) reported unmet need at the national level. Integrated analysis identified age, sex, and geographic setting associations with AT indicators, and explored how these factors intersect in relation to access barriers. Cost, limited awareness, sociocultural/environmental factors, and fragmented provision pathways emerged as the main constraints, with older adults, females, and those in rural and estate settings disproportionately affected. Embedding stakeholder engagement in this study enabled translation of findings into actionable recommendations to reduce financial barriers and strengthen provision systems.

**Implications of all the available evidence**

This study provides policy-relevant data to reduce unmet AT need in Sri Lanka. Improving access to AT will require coordinated action to address affordability, strengthen awareness systems, and decentralise and integrate service provision, while accounting for sociocultural/environmental factors. Available evidence highlights a need for life-course and equity-oriented approaches, as disparities correlate with intersecting factors and will likely widen with population ageing. Mixed-methods assessment combined with stakeholder engagement as used in this study offer a valuable approach for generating actionable evidence in data-sparse settings.

## Introduction

Assistive Technology (AT) encompasses a broad range of products, systems, and services designed to maintain or improve an individual’s functioning and independence, thereby promoting participation in education, employment, and community life.^1^ AT includes physical products such as wheelchairs, glasses, prosthetics, and hearing aids, as well as digital tools such as mobile phones and accessible software. Globally, the WHO estimates that over 2.5 billion people require AT, a figure that is projected to rise to 3.5 billion by 2050, driven by ageing populations and the increasing prevalence of non-communicable diseases. Yet only 10% of the population have access to the AT they need.^1^

With the highest proportion of older adults in South Asia (16.4% of its 22.16 million population aged 60 and above), Sri Lanka is facing accelerating demographic pressure on AT systems. While Sri Lanka has enacted progressive disability legislations, specific attention to AT within these frameworks remains limited.^2^ Evidence gaps further compound this policy deficit and no representative research has been conducted on AT access in the country. Further, diversity within disability and AT,^3^ as well as variation in study designs and target populations in similar research,^4^^,^^5^ limit the generalisability of external evidence and therefore its potential to inform AT policy development in Sri Lanka. Even in settings with adequate evidence, policy change is still challenged by a lack of stakeholder engagement during the research process,^6^ emphasising its crucial role in contextualising findings.^7^

To address these gaps, WHO validated tools were utilised to undertake a country capacity Assistive Technology Assessment (ATA-C),^8^ along with the rapid Assistive Technology Assessment (rATA) survey.^9^ The objective were to generate evidence and analyse AT indicators across key demographic groups; to understand barriers shaping access through lived experience and stakeholder perspectives; and to arrive at consensus on recommendations.

## Methods

This is a mixed methods study including a national household survey; key informant interviews and focus group discussions; and an adapted Delphi study consolidation workshop. To generate evidence on AT indicators—need, use, and unmet need—the rATA survey was administered between May and July 2024. Full details of the rATA protocol are available,^10^ in addition to the full questionnaire.^9^ In brief, the sampling procedure employed stratified random sampling with probability proportional-to-size selection across Sri Lanka’s 25 administrative districts to reflect urban (18%), rural (77%), and estate (4%) population distributions, based on the 2012 census. In Sri Lanka, ‘estates’ are large-scale agricultural lands that function as distinct residential/industrial communities. The proportionate household sample within each district stipulated the number of primary sampling units, which were Grama Niladhari Divisions (GNs) in this study. Within each GN, three random GPS points were generated, of which enumerators chose one that was residential as a starting point. From here, enumerators selected additional households using a skipping pattern tailored to urban, rural, and estate settings. All household members of the selected households (greater than 2 years of age) were eligible to participate. Proxy responses were permitted for children (<18 years) and individuals who were unable to self-report. Interviews were carried out in Sinhalese and Tamil by professional, locally based enumerators who received disability sensitivity and AT awareness training. The minimum required sample size for the quantitative survey was calculated as 1550 households, or 5735 individuals based on estimated access prevalence of 10%,^10^ non-response of 10%, average household size of 3.7, and all household members (>2 years) being considered eligible to participate.

The rATA questionnaire^9^ measures the prevalence of functional difficulty using the Washington Group Short Set^11^ and three key indicators of AT access in the population. AT indicators are defined based on WHO guidance. Use: Participant reports use of one or more assistive products. Unmet need: Participant reports need for one or more assistive products they do not currently have or must be replaced (regardless of use). Need: Participant reports use and/or unmet need.^1^^,^^10^

Individuals with need are considered to have access to AT if they do not report any unmet need.^10^ In this research, functional difficulty prevalence does not act as a direct proxy for disability prevalence but rather indicates a baseline population who could benefit from AT access. Gender was self-reported by all participants as male or female. Although the rATA included non-binary response options, no respondents selected these categories. Consequently, the variable is treated and reported as binary sex in statistical analysis.^12^ Ethnicity data are not standardly collected in rATA surveys. Survey data were weighted to account for sampling design and improve representation by the age, sex, and geographic distributions of each district from Sri Lanka’s 2012 census.

The ATA-C^8^ is a WHO tool designed to evaluate a country’s capacity to finance, regulate, procure, and provide AT developed by WHO with the intention to enable a system-level investigation. To carry out the ATA-C, qualitative research was undertaken between May and August 2024. Participants representing stakeholders in the AT sector in Sri Lanka were identified by the stakeholder groups and invited (by email or personal connections) to participate in this analysis through 26 key informant interviews and six focus group discussions. Participants were purposively sampled to represent the WHO 5Ps framework^1^ (People, Policy, Products, Provision, Personnel) and included government officials, service providers, AT users, and NGOs, sampled across ten districts: Colombo, Anuradhapura, Jaffna, Badulla, Galle, Ratnapura, Ampara, Batticaloa, Kalutara, and Gampaha. Interviews and discussions were set at locations chosen by the participants, with the majority conducted face-to-face (one online), and explored barriers to AT access, using a semi-structured topic guide. Focus groups lasted on average 83 min, whilst interviews lasted 34 min. The research team’s background was explained to participants (Appendix 4). An author familiar with the local context and trained in qualitative research (PJ) led the initial analysis, which was then shared with the wider research team to input and validate findings.

Data were audio-recorded, transcribed, and translated into English when necessary; and then analysed using reflexive thematic analysis (Braun and Clarke).^13^ Coding was iteratively refined through regular peer discussion and collaborative review sessions. Where empirical gaps were identified, additional interviews were conducted to ensure thematic saturation. Once structured, these findings were shared with participants in the Delphi workshop. The research team cross-referenced this thematic analysis with the rATA data to build a solid understanding of country AT need and capacity in Sri Lanka, to inform the next stage: the development of recommendations for action.

Stakeholders were involved throughout this research process, from its inception including key decision-making and evaluation stages, as recommended in the ATA-C guidelines. Once key themes were constructed, the research team convened for a two-day workshop in February 2025 to synthesise and validate preliminary findings from the quantitative and qualitative work combined, and test and evolve a consensus-based approach to recommendations, with over 30 stakeholders from diverse sectors including ministries, NGOs, OPDs, and academic partners. Employing an adapted Delphi methodology,^14^ the team tested an initial hypothesis of ten central recommendations—‘Hypothesis A’—built by the research team, against stakeholder expertise structuring the sessions around the ‘5P’ framework. Following inputs the team then revised the recommendations overnight to formulate ‘Hypothesis B,’ a revised set of recommendations which was then further and finally revised on the second day through plenary discussions on health system integration, local manufacturing, and high-level coordination mechanisms. The process culminated in a final set of core recommendations (‘Hypothesis C’) emerging from the research. STROBE,^15^ COREQ,^16^ and GRAMMS^17^ checklists were used to ensure appropriate reporting format (Appendix 1–3).

### Statistical analysis

Statistical analysis of the rATA survey included logistic regression to test associations between demographic factors (age, sex, urban/rural setting) with functional difficulty levels (any, high) and AT indicators (need, use, unmet need), and estimate predicted prevalences, both in/excluding glasses. Each factor was tested for association with each outcome individually (univariable) and all together (multivariable). Confidence intervals at 95% were calculated on the logit scale using model-based standard errors that accounted for sampling weights, stratification, and clustering at the household level, and then transformed back to the probability scale. Researchers accounted for clustering at the household level rather than PSU level, as PSU data were not made available for analysis due to data privacy restrictions. All survey analyses were done in R 4.4.0^18^ using the survey package.^19^

### Ethics statement

Ethical approval was granted by Sabaragamuwa University of Sri Lanka Faculty of Medicine Ethics Review Committee (ERC/FOM-SUSL/A 07-03-24) and University College London Computer Science Research Ethics Committee (UCL/CSREC/R/38-A1). Informed consent was obtained from all participants prior to involvement in the survey, interviews, focus groups, and workshops. Survey participants consented electronically via the survey tool while qualitative research participants provided written or verbal consent, depending on access requirements. All consent protocols detailed the study’s purpose, procedures, risks, and benefits, and explicitly stated participation was voluntary with the right to withdraw at any point during data collection.

### Role of the funding source

The funder of the study had no role in study design, data collection, data analysis, data interpretation, or writing of this article.

## Results

Of 1820 households sampled, 6106 individuals completed interviews, yielding a response rate of 93.6%. After removing records with any missing data, 6100 respondents were included in the analytic sample (Table 1). Participant ages ranged from 2 to 99; the median age was 39 years (IQR 19–57). Among the participants, 41.0% had any (“some” or more) difficulty, most often with vision (32.5%), mobility (16.9%), and remembering/cognition (12.6%). Further, 11.7% reported high functional difficulty (“a lot” or more). Including glasses as AT, 34.0% of the survey sample had AT need, 23.0% used AT, and 19.8% had unmet AT needs.

**Table 1:** Participant demographics from the rATA survey.

rATA survey sample (n = 6100)	Total (%)
Age groups	
2–9	612 (10.0%)
10–17	819 (13.4%)
18–29	890 (14.6%)
30–39	787 (12.9%)
40–49	912 (15.0%)
50–59	735 (12.0%)
60–69	725 (11.9%)
70–79	462 (7.6%)
80+	158 (2.6%)
Sex	
Female	3272 (53.6%)
Male	2828 (46.4%)
Setting	
Rural	4543 (74.5%)
Urban	1232 (20.2%)
Estate	325 (5.3%)
Any functional difficulty	
No	3600 (59.0%)
Yes	2500 (41.0%)
High functional difficulty	
No	5389 (88.3%)
Yes	711 (11.7%)
Vision difficulty	
Any level	1985 (32.5%)
None	4115 (67.5%)
Some	1591 (26.1%)
A lot	366 (6.0%)
Cannot do	28 (0.5%)
Hearing difficulty	
Any level	407 (6.7%)
None	5693 (93.3%)
Some	322 (5.3%)
A lot	77 (1.3%)
Cannot do	8 (0.1%)
Mobility difficulty	
Any level	1031 (16.9%)
None	5069 (83.1%)
Some	720 (11.8%)
A lot	275 (4.5%)
Cannot do	36 (0.6%)
Communication difficulty	
Any level	163 (2.7%)
None	5937 (97.3%)
Some	123 (2.0%)
A lot	31 (0.5%)
Cannot do	9 (0.1%)
Remembering/cognitive difficulty	
Any level	769 (12.6%)
None	5331 (87.4%)
Some	671 (11.0%)
A lot	87 (1.4%)
Cannot do	11 (0.2%)
Self-care difficulty	
Any level	457 (7.5%)
None	5643 (92.5%)
Some	303 (5.0%)
A lot	120 (2.0%)
Cannot do	34 (0.6%)
Need (including glasses)	
No	4024 (66.0%)
Yes	2076 (34.0%)
Use (including glasses)	
No	4696 (77.0%)
Yes	1404 (23.0%)
Unmet need (including glasses)	
No	4892 (80.2%)
Yes	1208 (19.8%)
Need (excluding glasses)	
No	5441 (89.2%)
Yes	659 (10.8%)
Use (excluding glasses)	
No	5773 (94.6)
Yes	327 (5.4%)
Unmet need (excluding glasses)	
No	5641 (92.5%)
Yes	459 (7.5%)

Young age groups (2–9 and 10–17) are used to show the number and percent of children included; analyses group ages < 18 years.

When including glasses, females had slightly higher odds of unmet need (OR 1.16, 95% CI 1.02–1.33) (Table 2) compared with males in the multivariable model. This association was stronger when excluding glasses in univariable (OR 1.36, 1.11–1.67) and multivariable models (OR 1.41, 1.15–1.73) (Appendix 5). Increasing age was consistently associated with higher odds of reporting any and high levels of functional difficulty, need, use, and unmet need, in both models, and when including and excluding glasses. Compared with urban settings, rural and estate had similar odds of both functional difficulty levels and unmet need, yet lower odds of need and use, in both models (Table 2, Appendix 5). When excluding glasses, only estate settings had lower odds of AT use compared to urban.

**Table 2:** Odds ratios for functional difficulty and AT indicators, including glasses.

	Univariable OR (95% CI)	Multivariable OR (95% CI)
Any functional difficulty		
Sex		
Male	1.00	1.00
Female	1.03 (0.92–1.15)	1.07 (0.97–1.19)
Age		
<18	1.00	1.00
18–64	2.20^c^ (1.90–2.56)	2.20^c^ (1.90–2.55)
65+	10.14^c^ (8.12–12.68)	10.08^c^ (8.07–12.59)
Setting		
Urban	1.00	1.00
Rural	0.87 (0.75–1.01)	0.90 (0.77–1.05)
Estate	0.99 (0.74–1.32)	0.94 (0.71–1.25)
High functional difficulty		
Sex		
Male	1.00	1.00
Female	1.00 (0.84–1.19)	1.05 (0.89–1.24)
Age		
<18	1.00	1.00
18–64	2.39^c^ (1.82–3.14)	2.41^c^ (1.83–3.16)
65+	8.70^c^ (6.40–11.83)	8.75^c^ (6.45–11.88)
Setting		
Urban	1.00	1.00
Rural	1.06 (0.84–1.33)	1.09 (0.87–1.37)
Estate	0.83 (0.48–1.43)	0.80 (0.45–1.41)
Need (including glasses)		
Sex		
Male	1.00	1.00
Female	1.01 (0.90–1.13)	1.05 (0.94–1.18)
Age		
<18	1.00	1.00
18–64	2.30^c^ (1.96–2.70)	2.30^c^ (1.96–2.70)
65+	8.79^c^ (7.08–10.90)	8.73^c^ (7.04–10.81)
Setting		
Urban	1.00	1.00
Rural	0.79^b^ (0.67–0.93)	0.83^a^ (0.71–0.98)
Estate	0.66^a^ (0.47–0.91)	0.65^b^ (0.47–0.89)
Use (including glasses)		
Sex		
Male	1.00	1.00
Female	0.93 (0.81–1.06)	0.98 (0.86–1.11)
Age		
<18	1.00	1.00
18–64	2.68^c^ (2.19–3.29)	2.69^c^ (2.19–3.29)
65+	8.05^c^ (6.30–10.28)	7.92^c^ (6.21–10.10)
Setting		
Urban	1.00	1.00
Rural	0.74^b^ (0.62–0.89)	0.78^b^ (0.65–0.94)
Estate	0.46^c^ (0.31–0.69)	0.46^c^ (0.31–0.68)
Unmet need (including glasses)		
Sex		
Male	1.00	1.00
Female	1.13 (0.98–1.29)	1.16^a^ (1.02–1.33)
Age		
<18	1.00	1.00
18–64	1.84^c^ (1.52–2.22)	1.84^c^ (1.52–2.22)
65+	5.09^c^ (4.03–6.42)	5.12^c^ (4.06–6.45)
Setting		
Urban	1.00	1.00
Rural	1.01 (0.83–1.22)	1.03 (0.85–1.25)
Estate	1.12 (0.79–1.60)	1.08 (0.75–1.54)

Odds ratio (OR), confidence interval (CI).

Logistic regression results are presented odds ratios for univariable (single) and multivariable (joint) models, testing the association of all demographic factors (first individually, then all together) with each AT indicator (need, use, unmet need) and functional difficulty level (any, high), including glasses as AT, using weighted survey data.

a p < 0.05.

b p < 0.01.

c p < 0.001.

The overall predicted prevalence of any functional difficulty and AT need (including glasses) was estimated at 35.2% (33.9–36.6) and 28.4% (27.1–29.8), respectively (Table 3). There was no estimated sex or setting difference in the prevalence of either functional difficulty level. Both level and all indicator prevalences increased significantly with age, with an estimated 73.7% (70.4–76.8) of adults older than 65 years experiencing some or more difficulty (Table 3). When excluding glasses, females had a slightly higher prevalence of unmet need (6.7%, 5.8–7.7) compared to males (4.8%, 4.1–5.7), and estate settings reported lower use (1.2%, 0.5–3.0) than rural (3.8%, 3.2–4.5) and urban (4.6%, 3.3–6.5) (Appendix 6).

**Table 3:** Predicted prevalence of functional difficulty and AT indicators, including glasses.

	Any functional difficulty	High functional difficulty	Need (including glasses)	Use (including glasses)	Unmet need (including glasses)
	Prev % (95% CI)	Prev % (95% CI)	Prev % (95% CI)	Prev % (95% CI)	Prev % (95% CI)
Overall	35.2% (33.9–36.6)	8.5% (7.7–9.3)	28.4% (27.1–29.8)	18.1% (16.9–19.3)	16.4% (15.4–17.6)
Sex					
Male	34.4% (32.5–36.3)	8.3% (7.3–9.4)	27.9% (26.1–29.7)	18.3% (16.8–19.9)	15.4% (14.1–16.8)
Female	36.0% (34.2–38.0)	8.6% (7.7–9.7)	28.9% (27.2–30.8)	17.8% (16.3–19.4)	17.5% (16.0–19.0)
Age					
<18	21.7% (19.6–24.1)	4.3% (3.4–5.5)	16.6% (14.7–18.7)	9.2% (7.7–10.9)	10.6% (9.1–12.4)
18–64	37.9% (36.3–39.6)	9.8% (8.9–10.9)	31.5% (29.9–33.1)	21.4% (20.0–22.9)	17.9% (16.6–19.2)
65+	73.7% (70.4–76.8)	28.4% (25.0–32.0)	63.6% (60.0–67.0)	44.7% (41.1–48.4)	37.8% (34.2–41.5)
Setting					
Urban	37.2% (34.1–40.5)	8.1% (6.7–9.8)	32.2% (29.2–35.4)	22.1% (19.4–25.0)	16.1% (13.8–18.7)
Rural	34.7% (33.1–36.3)	8.8% (7.9–9.8)	28.0% (26.4–29.5)	17.8% (16.5–19.2)	16.5% (15.3–17.8)
Estate	35.7% (30.0–41.9)	6.4% (4.0–10.3)	22.8% (18.1–28.3)	10.9% (7.8–15.1)	17.1% (13.2–22.0)

Confidence interval (CI); Predicted prevalence (Prev). Predicted prevalences and 95% CIs are presented for weighted survey data including glasses and aggregated by demographic factors. Prevalence estimates are given with 95% confidence intervals in parentheses.

**Fig. 1:**
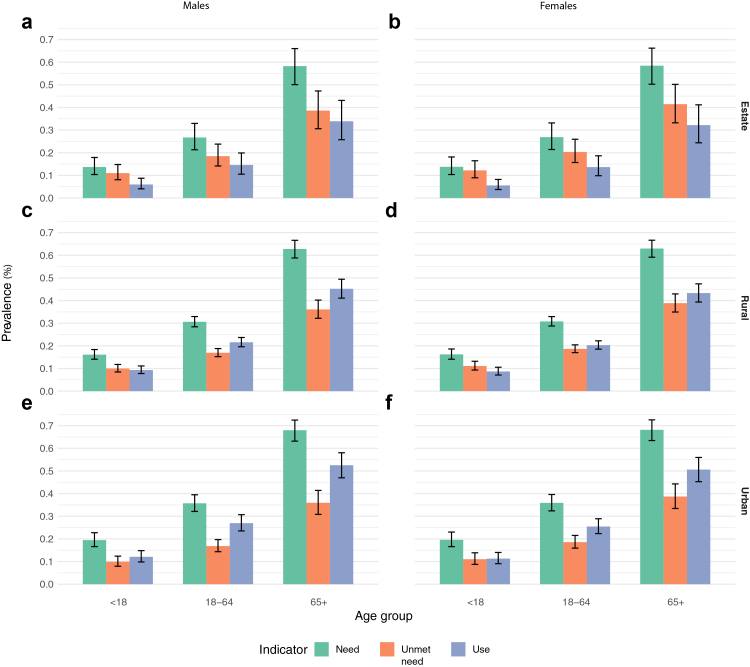
**Disaggregated predicted prevalence for AT indicators (use, need, and unmet need), including glasses.** Predicted prevalences and 95% CIs are presented for AT indicators disaggregated by age, sex, and setting, from weighted survey data including glasses. Prevalence estimates are represented by the y-axis (%), indicators are specified by bar colours (need in green, unmet need in orange, use in blue), and 95% CIs are presented as error bars. Bars ascend in increasing age group order. Figure panels represent groups in this order: a) estate males; b) estate females; c) rural males; d) rural females; e) urban males; f) urban females.

Stratifying indicators by all demographic factors revealed similar patterns of age-related increase in all indicators, by both sex and setting (Fig. 1). Use was more prevalent than unmet need for urban males and females between 18 and 64 years. When excluding glasses, similar rates between sex and the age-related pattern persisted, however rates of unmet need were higher than use among urban females (<18 years), rural females (all age groups) and estate females (>18 years (Appendix Fig. S1)).

Eighty-seven individuals participated in focus group discussions and key informant interviews, representing one (and often more) identities/sectors set out in the ‘5P’ framework. Thirty-seven (43%) were female, with 57% (n = 50) from the Sinhala population and 24% (n = 21) from the Tamil population. Nine participants did not disclose their ethnicity. The present section organises the insight derived across these personal and professional perspectives in terms of systemic barriers to AT access along with illustrative quotes.

### Cost

High cost emerged as a prominent barrier, compounded by limited government support and reliance on out-of-pocket expenditure. While some assistance exists for low-income households, the process was widely described as slow, complex, and inefficient.

Although many non-specialised AT are available in urban settings, prices rose following the 2022 economic crisis. One participant’s experience showed how the increasing cost and availability of products can be prohibitive: *“The hearing aid that was 60,000 LKR around five or [so] years back is now about 100,000 LKR to 200,000 LKR or more. Prices are doubled. So, then there’s a huge problem.”*

Participants often found travel to access AT too costly: One described how there is a lack of public transportation connecting their village with the city, but they found it expensive to hire a taxi to obtain AT and attend appointments: *“… there should be better transport facilities, I think … There are two buses to Galle, but it’s better if there are some transport facilities to [my village] in Hikkaduwa as well. So, it costs around 300 LKR to go for appointments by three-wheeler [taxi]. So, if there’s a bus, that 300 LKR could be saved.”*

Repair and replacement services were largely unavailable or unaffordable, forcing users to adapt or abandon products. The cost of consumables, such as hearing aid batteries, was a recurrent concern.

Most participants accessed AT through charity programs that provide free assistive products, with religious institutions, local and international non-governmental organisations (NGOs), community organisations, and philanthropy noted as main sources. Though some community organisations attempt to provide support and AT systematically and consistently, most charity offerings are one-off. These organisations depend heavily on international donors, which can be inconsistent.

One participant shared that managing donations often requires more work once received: *“We receive donations as well. And we check whether those are what we can distribute because some give us the used ones [assistive products]. So, we can’t take those types of ones. We anyway don’t get reading glasses. We got some frames from Australia. So, we get the useable frames, and then they put lenses, and our suppliers fix those things.”*

While participants expressed gratitude for these donations, they often noted these AT are not suitable: *“… sometimes they [charity organizations] give, but these spectacles are not really productive. They just give them for the sake of distributing them.”*

### Awareness and information gaps

Awareness of AT was generally low, particularly in rural and estate communities. There is no centralised mechanism for disseminating information, leaving users dependent on peers or NGOs. One participant said they only became aware of useful digital AT when they came to Colombo for further education. They had no access to any information about digital AT when they lived in a rural village. *“… I learned some techniques from my friends at the school … Then, I learned more during my university years … I discovered Google Assistant on my own.”* Caregivers, who can play a critical role in enabling AT users’ access, also often lack knowledge about available AT.

Some participants found information on accessing government-funded AT was confusing or inaccessible. Participants described having to repeatedly chase local officials to progress their applications for AT. Participants said that, even if they knew or had a personal relationship with the correct official, it would be challenging to promptly get the AT they needed. For instance: *“… We used to contact the social service officer by obtaining his mobile number after some effort and send the name list [of assistive devices].”*

The overall absence of regulations for AT development and provision was highlighted by a participant: *“We should have the standards, but sometimes the standard becomes an obstacle for development. As an example, for a hearing aid, there are standards and guidelines. But, to follow that guideline, you have to have that technology. So, we can’t manufacture it locally. I agree that we should have standards and regulations. But in developing them, we should also look at the country’s conditions and requirements.”*

After-sales services like repair are limited in Sri Lanka, and correspondingly, awareness of these sparsely available services was low among AT users. One participant mentioned that because they could not access repair services, the quality of their product was reduced: *“… since it couldn’t be repaired earlier, now its hearing has gotten deteriorated. It’s not broken, but I hear sounds with a filter, like birds chirping.”*

### Social, cultural and environmental factors

Stigma was identified as a pervasive barrier, especially for hearing aids and mobility products. Participants reported that negative societal attitudes discouraged AT use, reinforcing feelings of dependency and exclusion.

One participant said, *“After purchasing the hearing equipment, I wore it for a few days. Then, others made fun of me … saying I am deaf. Then I stopped using it altogether because of the ridicule.”* This same participant also felt that, because of this ridicule, they would never be able to marry. They added: *“… some people feel ashamed when wearing assistive devices. When they face society, they worry that others might perceive them as having problems. This attitude issue still exists in society …”*

This stereotype has added socio-economic consequences, leading individuals and their families to believe they should not take advantage of available opportunities. Inaccessible environments, including transport, government offices, and banking services, further limited the effectiveness of AT and entrenched harmful beliefs.

### Provision and coordination challenges

Whilst government provision pathways were well-established, they were reportedly fragmented and faced many challenges in coordination and resource allocation with overlapping roles and weak coordination between key ministries. While public officers are stationed from national to local levels to facilitate AT access, the system suffers from chronic underfunding and a lack of integration between health and social services. One participant stated, in reference to government: *“… Inter-relationship is very less because health sector would only know health sector and social services would only know social service. It is not helping.”*

Provision of services follows a tripartite model involving government welfare, NGOs, and private out-of-pocket expenses. However, government support is strictly means-tested, often excluding the “missing middle” who exceed low-income thresholds but cannot afford private AT. One participant shared that at least 5000 students are identified annually as needing AT. However, delays in government provision can reduce their functional capacity and disrupt their education: *“When they [students] need to start school, they are delayed as they don’t have provision [of AT].”*

After integrating survey and qualitative findings, the consensus building workshop refined the hypotheses of the research team to a final list of ten recommendations (Table 4) responding to demographic inequities, lived experiences of barriers, and stakeholder input.

**Table 4:** Recommendations for improving AT access in Sri Lanka.

Recommendation	Core Actions
1. Reduce Product Cost	Pooled procurement; set standards; incentivize local production; tax exemptions; consider ceiling prices; explore user allowances
2. Enable Better Coordination	Establish AT focal point under President/PM; ministry-level focal points; create national AT network
3. Empower AT Users & Reduce Stigma	Implement Disability Policy; revise Disability Act; promote education & employment; awareness campaigns
4. Support AT Training	Roll out WHO Training in Assistive Products (TAP) for frontline workers; advanced training via NVQ/SLQF; set up regional labs
5. Gather Better Data & Evidence	Digital data system; integrate AT questions in census; regular data sharing; monitor policy impact
6. Grow AT Innovation Sector	Create innovation hubs; digital info platform; support start-ups; research symposiums
7. Pilot Community Hubs (Older Adults)	Local AT centres; repair & personalization services; train volunteers; digital skills for seniors
8. Enable AT Access for Young People	Embed universal design in education; ensure accommodations; screening & therapy in schools; parent training
9. Improve Accessibility of Physical & Digital Spaces	Enforce accessibility laws; enhance digital banking; improve transport infrastructure
10. Create AT Program within Health System	Map provision; establish technical group; integrate AT into pooled procurement; include AT in health strategy

## Discussion

This mixed-methods study included 6100 surveyed individuals and 87 participants in qualitative interviews and discussions, identifying high unmet AT need and multiple access barriers in Sri Lanka. Although 35.2% (33.9–36.6) of the population is estimated to experience functional difficulty, 18.1% (16.9–19.3) use AT, and 16.4% (15.4–17.6) have unmet need, highlighting systemic gaps. Age, sex, and setting further influence access. Qualitative findings attribute barriers to cost, limited awareness, sociocultural and environmental factors, and coordination challenges. Combined analysis enables a deeper understanding of how barriers restrict equitable access across groups. The integrated approach also informed targeted recommendations, stakeholder commitments, and policy change.

Age had the most reliable association with AT indicators in this study. Any/high functional difficulty and AT need increased substantially with age. Although AT use was also highest among older adults, levels of unmet need remained prevalent, indicating that current systems only partially meet demand, and motivating the explicit inclusion of older adults in community AT hub services (Recommendation 7). These findings are also consistent with global,^1^^,^^20^^,^^21^ and national^2^^,^^22^ evidence that functional difficulty and AT indicators increase with age. Quantitative and qualitative findings also point to critical access gaps among younger populations, particularly in rural/estate settings, and include limited awareness of emerging AT and delays in school-based referral pathways. Considering the impact of AT on health, social, and economic outcomes,^1^ early-life access gaps forecast late-life consequences and outline the importance of school-based screening/referral programmes (Recommendation 8). In addition to meeting the support needs of the older population, investment in AT access at younger ages should also be considered as an investment in the population’s long-term health planning (Recommendation 10). The (repeated) administration of the rATA can support this aim—as an international standard for data collection—through monitoring AT access across the life course.

Sex differences in AT indicators were more pronounced when excluding glasses as AT. Despite no evident differences in functional difficulty or AT need, compared to males, females residing in urban (<18 years), rural (all age groups), and estate settings (>18 years) had higher estimated prevalences of unmet need for non-glasses AT. This gap suggests sociocultural/environmental factors identified as access barriers in qualitative analysis may be experienced differently across sex and setting, highlighting the gendered impact of stigma and awareness on AT access, and leading to a recommendation dedicated to both (Recommendation 3). These findings are consistent with regional^23^^,^^24^ and global^1^ evidence identifying better access among males. For example, Senjam et al.’s administration of the rATA survey in India reported females were more likely to need AT, less likely to use it, and more likely to report unmet need compared to males (including glasses).^23^ This pattern is also evidenced in the GReAT, reporting sex-disparities in access that widen with decreasing HDI score.^1^ Notably, males and females reported similar levels of functional difficulty in the present study, contrasting with both Sri Lanka’s 2012 census, which reports that 57.0% of those with functional difficulty were females,^22^ and global patterns of higher functional difficulty^21^ (and unmet AT need^1^^,^^25^) among females. The present finding may partly reflect the long-term impacts of Sri Lanka’s civil war, which could have affected current disability prevalence among males currently at mid- and later life.^26^ As inclusion or exclusion of glasses as AT produced mixed effects of sex on AT indicators, the present analysis emphasised the importance of sex-disaggregated and product-disaggregated monitoring, and gender-sensitive approaches to AT provision (Recommendations 4 and 5).^1^^,^^25^ These challenges are amplified at demographic intersections; for example, cost emerged as a key barrier in the qualitative investigation (Recommendation 1), and females (particularly in older age) are more likely to experience poverty,^27^ increasing their risk of unmet AT need. These inequities occur across the life course, leading to compounded disadvantage in later life among females. Ageing-related AT needs must therefore be understood in relation to gendered life trajectories, disability patterns, and unequal access to resources across the life course.

Geographic inequities in access were evident across settings. Despite similar odds of reporting functional difficulty and lower odds of use, rural and estate residents were not more likely to report unmet need, indicating a gap in perceived benefit or awareness of AT outside of urban areas. Better access among urban residents is consistent with evidence from Nepal, India, and Bangladesh.^28^ Disaggregated predicted prevalences identified higher rates of unmet need among females in rural and estate setting. These results point to structural access gaps in underserved communities, particularly estate communities. Relevant barriers include limited access to information, given there is no centralised mechanism for dissemination (Recommendation 2); reduced availability of specialised services in non-urban areas (Recommendation 6); and prohibitively expensive and inaccessible transport from remote locations (Recommendation 9). The present study highlights the importance of decentralised AT service delivery, outreach and mobile services, and targeted subsidies to address issues of coordination failures and delayed access (Recommendation 7).

This study presents findings from a population-based survey and qualitative data collection on AT access in Sri Lanka, along with a Delphi consensus-building workshop to arrive at actionable recommendations. This work helps to bridge an evidence gap in this sector, with two recommendations now adopted at policy-level in Sri Lanka; specifically, and has preceeded the exemption of custom duties on imported assistive products^29^ and the expansion of the formal access mechanism and individual budget for AT purchasing^30^ (Recommendations 1 and 3).

Several limitations should be noted. Enumerating people with disabilities in household surveys is challenging, and individuals in institutional settings were not included in the quantitative sample, although this group is likely small in Sri Lanka. These findings likely underrepresent true AT need, use, and unmet need in Sri Lanka. rATA data were not reported by ethnicity, which may miss important inequities. Proxy reporting was permitted in some cases, which may introduce inaccuracies. AT stakeholders were diverse, and although efforts were made to ensure representation across demographics and roles, their adequacy is difficult to assess. Participants may not reflect the most marginalised groups, and social desirability bias may have influenced reporting of AT indicators as well as perspectives from professional or government representatives. Qualitative quotes were not linked to demographic data to preserve consistent anonymisation, which may limit group-specific interpretation. Qualitative data were collected in 10 of 25 districts and are not nationally representative, which introduces geographic asymmetry with the nationally representative rATA, and limits both the transferability of findings (particularly for the estate sector) and the direct integration of those datasets. Indeed, integrating quantitative and qualitative findings is inherently interpretive, and different researchers may reach different conclusions. This limitation was mitigated through structured, iterative feedback within the Delphi process. Updating the rATA questionnaire to utilise the Washington Group Extended Set would also have enabled better inclusion of individuals with psychosocial disabilities in the household survey. The protections around PSU data prevented analysis by this clustering level, and while household clustering was accounted for, variance may still be underestimated. These findings are specific to Sri Lanka and may not be generalised to other contexts due to country-specific AT systems/policies and population characteristics. The concluding stakeholder workshop’s effectiveness at garnering commitments was facilitated by consistent engagement opportunities built into the assessment approach, as well as an existing government commitment to addressing AT needs, which may indicate a particularly supportive policy environment. Whether resulting changes lead to sustained impact remains uncertain, suggesting multi-year follow-up to evaluate outcomes as an important area of future research.

This study identified age, sex, and setting to be associated with AT access indicators; and cost, awareness, sociocultural/environmental factors, and coordination failures as primary barriers to AT use in Sri Lanka. Combined analysis suggested intersectional factors increased the risk of facing access barriers, particularly for women in rural and estate areas. With the forecasted increase in the proportion of older population in Sri Lanka and globally, there is a probability of widening AT access gaps. Governments should formulate policies to ensure AT is available to all who need it, with tailored service provision for groups that tend to have more limited access. Monitoring the effectiveness of these policies at population-level can be achieved using validated tools in routine national data collection and longitudinal surveys, while mixed methods approaches can further help to shape equity-oriented policy planning.

## Contributors

DP and VA conceived the study. DP, JD, MK, and VA devised the study methodology. DP, PJ, HW, ND, and VA did the investigation. DP, PJ, JD, HW, ND directly accessed and verified the underlying data. DP, PJ, and JD did the formal data analysis. JD curated the data. DP and JD and PJ wrote the first draft of the manuscript. PJ, HW, ND, MK, and VA reviewed and edited the manuscript. JD visualised the data. VA and MK supervised the study. VA acquired the study funding. JD had final responsibility for the decision to submit for publication. All authors have read and approved the final version of the manuscript.

## Data sharing statement

All study data are managed by the project funder and may be made available upon request.

## Declaration of interests

All authors declare no competing interests.
