# Supplementary material for: Assistive technology unmet need and barriers to access in Sri Lanka: a mixed-methods study (2024)
Source: Lancet Reg Health Southeast Asia. 2026 Jun 24;51:100807. doi: 10.1016/j.lansea.2026.100807 (PMC13320440; doi:10.1016/j.lansea.2026.100807)
Supplement: Appendix 1–6 [file mmc1.docx]

**Appendix**

Contents

[**Table 1: Strengthening the Reporting of Observational Studies in Epidemiology (STROBE) checklist for cross-sectional studies** 2](#_Toc229170631)

[**Table 2: Consolidated criteria for reporting qualitative studies (COREQ)** 4](#_Toc229170632)

[**Table 3: Good Reporting of a Mixed Methods Study (GRAMMS) guidelines** 6](#_Toc229170633)

[**Table 5: Odds ratios for functional difficulty and AT indicators, excluding glasses** 8](#_Toc229170634)

[**Table 6**: **Aggregated predicted prevalence for AT indicators, excluding glasses** 9](#_Toc229170635)

[**Figure 1: Disaggregated predicted prevalence for AT indicators, excluding glasses** 10](#_Toc229170636)

# **Table 1: Strengthening the Reporting of Observational Studies in Epidemiology (STROBE) checklist for cross-sectional studies**

|  | **Item Description** | **Section, paragraph number  (or reason for not reporting)** |
| --- | --- | --- |
| **Title and abstract** | | |
| [1a. Indicate the study’s design](https:/resources.equator-network.org/reporting-guidelines/strobe/items/title-abstract-indicate-study-design.html?utm_source=strobe&utm_medium=checklist&utm_campaign=1_1) | Indicate the study’s design with a commonly used term in the title or the abstract. | Title |
| [1b. Abstract](https:/resources.equator-network.org/reporting-guidelines/strobe/items/abstract.html?utm_source=strobe&utm_medium=checklist&utm_campaign=1_1) | Provide in the abstract an informative and balanced summary of what was done and what was found. | Abstract, p3 |
| **Introduction** |  |  |
| [2. Background / rationale](https:/resources.equator-network.org/reporting-guidelines/strobe/items/background-rationale.html?utm_source=strobe&utm_medium=checklist&utm_campaign=1_1) | Explain the scientific background and rationale for the investigation being reported. | Introduction, p2 |
| [3. Objectives](https:/resources.equator-network.org/reporting-guidelines/strobe/items/objectives.html?utm_source=strobe&utm_medium=checklist&utm_campaign=1_1) | State specific objectives, including any prespecified hypotheses. | Introduction, p3 |
| **Methods** | | |
| [4. Study design](https:/resources.equator-network.org/reporting-guidelines/strobe/items/study-design.html?utm_source=strobe&utm_medium=checklist&utm_campaign=1_1) | Present key elements of study design early in the paper. | Methods, p1-p4 |
| [5. Setting](https:/resources.equator-network.org/reporting-guidelines/strobe/items/setting.html?utm_source=strobe&utm_medium=checklist&utm_campaign=1_1) | Describe the setting, locations, and relevant dates, including periods of recruitment, exposure, follow-up, and data collection. | Methods, p2 |
| [6a. Eligibility criteria](https:/resources.equator-network.org/reporting-guidelines/strobe/items/eligibility-criteria.html?utm_source=strobe&utm_medium=checklist&utm_campaign=1_1) | **Cross-sectional study:** Give the eligibility criteria, and the sources and methods of selection of participants. | Methods, p2 |
| [7. Variables](https:/resources.equator-network.org/reporting-guidelines/strobe/items/variables.html?utm_source=strobe&utm_medium=checklist&utm_campaign=1_1) | Clearly define all outcomes, exposures, predictors, potential confounders, and effect modifiers. Give diagnostic criteria, if applicable. | Methods, p1, p3 |
| [8. Data sources / measurement](https:/resources.equator-network.org/reporting-guidelines/strobe/items/data-sources-measurement.html?utm_source=strobe&utm_medium=checklist&utm_campaign=1_1) | For each variable of interest give sources of data and details of methods of assessment (measurement). Describe comparability of assessment methods if there is more than one group. | Methods, p1-p3 |
| [9. Bias](https:/resources.equator-network.org/reporting-guidelines/strobe/items/bias.html?utm_source=strobe&utm_medium=checklist&utm_campaign=1_1) | Describe any efforts to address potential sources of bias. | Methods, p2, p5 |
| [10. Study size](https:/resources.equator-network.org/reporting-guidelines/strobe/items/study-size.html?utm_source=strobe&utm_medium=checklist&utm_campaign=1_1) | Explain how the study size was arrived at. | Methods, p2 |
| [11. Quantitative variables](https:/resources.equator-network.org/reporting-guidelines/strobe/items/quantitative-variables.html?utm_source=strobe&utm_medium=checklist&utm_campaign=1_1) | Explain how quantitative variables were handled in the analyses. If applicable, describe which groupings were chosen, and why. | Methods, p1, p3 |
| [12a. Statistical methods](https:/resources.equator-network.org/reporting-guidelines/strobe/items/statistical-methods-description.html?utm_source=strobe&utm_medium=checklist&utm_campaign=1_1) | Describe all statistical methods, including those used to control for confounding. | Methods, p4 |
| [12b. Statistical methods – subgroups and interactions](https:/resources.equator-network.org/reporting-guidelines/strobe/items/statistical-methods-subgroups-interactions.html?utm_source=strobe&utm_medium=checklist&utm_campaign=1_1) | Describe any methods used to examine subgroups and interactions. | Methods, p5 |
| [12c. Statistical methods – missing data](https:/resources.equator-network.org/reporting-guidelines/strobe/items/statistical-methods-missing-data.html?utm_source=strobe&utm_medium=checklist&utm_campaign=1_1) | Explain how missing data were addressed. | Results, p1 |
| [12diii. Statistical methods – sampling strategy](https:/resources.equator-network.org/reporting-guidelines/strobe/items/statistical-methods-analytical-methods-sampling-strategy.html?utm_source=strobe&utm_medium=checklist&utm_campaign=1_1) | **Cross-sectional study:** If applicable, describe analytical methods taking account of sampling strategy. | Methods, p4 |
| [12e. Statistical methods – sensitivity analyses](https:/resources.equator-network.org/reporting-guidelines/strobe/items/statistical-methods-sensitivity-analyses.html?utm_source=strobe&utm_medium=checklist&utm_campaign=1_1) | Describe any sensitivity analyses. | N/A |
| **Results** | | |
| [13a. Participant numbers](https:/resources.equator-network.org/reporting-guidelines/strobe/items/participants-numbers.html?utm_source=strobe&utm_medium=checklist&utm_campaign=1_1) | Report the numbers of individuals at each stage of the study—e.g., numbers potentially eligible, examined for eligibility, confirmed eligible, included in the study, completing follow-up, and analysed; Consider use of a flow diagram. | Results, p1 |
| [13b. Participants – non-participation](https:/resources.equator-network.org/reporting-guidelines/strobe/items/participants-non-participation.html?utm_source=strobe&utm_medium=checklist&utm_campaign=1_1) | Give reasons for non-participation at each stage. | N/A |
| [13c. Participants – flow diagram](https:/resources.equator-network.org/reporting-guidelines/strobe/items/participants-flow-diagram.html?utm_source=strobe&utm_medium=checklist&utm_campaign=1_1) | Consider use of a flow diagram. | N/A |
| [14a. Descriptive data – participant characteristics](https:/resources.equator-network.org/reporting-guidelines/strobe/items/descriptive-data-participant-characteristics.html?utm_source=strobe&utm_medium=checklist&utm_campaign=1_1) | Give characteristics of study participants (e.g., demographic, clinical, social) and information on exposures and potential confounders. Present the information in a table. | Results, p1, Table 1 |
| [14b. Descriptive data – missing data](https:/resources.equator-network.org/reporting-guidelines/strobe/items/descriptive-data-missing-data.html?utm_source=strobe&utm_medium=checklist&utm_campaign=1_1) | Indicate the number of participants with missing data for each variable of interest. | Results, p1 |
| [16a. Main results](https:/resources.equator-network.org/reporting-guidelines/strobe/items/main-results.html?utm_source=strobe&utm_medium=checklist&utm_campaign=1_1) | Give unadjusted estimates and, if applicable, confounder-adjusted estimates and their precision (e.g., 95% confidence intervals). Make clear which confounders were adjusted for and why they were included. | Results, p2, p3, Table 2 |
| [16b. Main results – category boundaries](https:/resources.equator-network.org/reporting-guidelines/strobe/items/main-results-category-boundaries.html?utm_source=strobe&utm_medium=checklist&utm_campaign=1_1) | Report category boundaries when continuous variables were categorised. | Results, p1 |
| [16c. Main results – risk](https:/resources.equator-network.org/reporting-guidelines/strobe/items/main-results-risk.html?utm_source=strobe&utm_medium=checklist&utm_campaign=1_1) | If relevant, consider translating estimates of relative risk into absolute risk for a meaningful time period. | N/A |
| [17. Other analyses](https:/resources.equator-network.org/reporting-guidelines/strobe/items/other-analyses.html?utm_source=strobe&utm_medium=checklist&utm_campaign=1_1) | Report other analyses done—e.g., analyses of subgroups and interactions, and sensitivity analyses. | N/A |
| **Discussion** | | |
| [18. Key results](https:/resources.equator-network.org/reporting-guidelines/strobe/items/key-results.html?utm_source=strobe&utm_medium=checklist&utm_campaign=1_1) | Summarise key results with reference to study objectives. | Discussion, p1 |
| [19. Limitations](https:/resources.equator-network.org/reporting-guidelines/strobe/items/limitations.html?utm_source=strobe&utm_medium=checklist&utm_campaign=1_1) | Discuss limitations of the study, taking into account sources of potential bias or imprecision. Discuss both direction and magnitude of any potential bias. | Discussion, p6 |
| [20. Interpretation](https:/resources.equator-network.org/reporting-guidelines/strobe/items/interpretation.html?utm_source=strobe&utm_medium=checklist&utm_campaign=1_1) | Give a cautious overall interpretation considering objectives, limitations, multiplicity of analyses, results from similar studies, and other relevant evidence. | Discussion, p2-p5 |
| [21. Generalisability](https:/resources.equator-network.org/reporting-guidelines/strobe/items/generalisability.html?utm_source=strobe&utm_medium=checklist&utm_campaign=1_1) | Discuss the generalisability (external validity) of the study results. | Discussion, p6, p7 |
| **Other information** | | |
| [22. Funding](https:/resources.equator-network.org/reporting-guidelines/strobe/items/funding.html?utm_source=strobe&utm_medium=checklist&utm_campaign=1_1) | Give the source of funding and the role of the funders for the present study and, if applicable, for the original study on which the present article is based. | Methods, p8 |

# **Table 2: Consolidated criteria for reporting qualitative studies (COREQ)**

| **No. Item** | **Guide questions/description** | **Section, paragraph number** |
| --- | --- | --- |
| **Domain 1: Research team and reﬂexivity** |  |  |
| *Personal Characteristics* |  |  |
| 1. Inter viewer/facilitator | Which author/s conducted the interview or focus group? | Methods, p6 |
| 2. Credentials | What were the researcher’s credentials? E.g. PhD, MD | Methods, p6; Appendix Table 4 |
| 3. Occupation | What was their occupation at the time of the study? | Appendix Table 4 |
| 4. Gender | Was the researcher male or female? | Appendix Table 4 |
| 5. Experience and training | What experience or training did the researcher have? | Methods, p6; Appendix Table 4 |
| *Relationship with participants* |  |  |
| 6. Relationship established | Was a relationship established prior to study commencement? | N/A |
| 7. Participant knowledge of the interviewer | What did the participants know about the researcher? e.g. personal goals, reasons for doing the research | Methods, p6; Appendix Table 4 |
| 8. Interviewer characteristics | What characteristics were reported about the inter viewer/facilitator? e.g. Bias, assumptions, reasons and interests in the research topic | Methods, p6; Appendix Table 4 |
| **Domain 2: Study design** |  |  |
| *Theoretical framework* |  |  |
| 9. Methodological orientation and Theory | What methodological orientation was stated to underpin the study? e.g. grounded theory, discourse analysis, ethnography, phenomenology, content analysis | Methods, p7 |
| *Participant selection* |  |  |
| 10. Sampling | How were participants selected? e.g. purposive, convenience, consecutive, snowball | Methods, p6 |
| 11. Method of approach | How were participants approached? e.g. face-to-face, telephone, mail, email | Methods, p6 |
| 12. Sample size | How many participants were in the study? | Results, p5 |
| 13. Non-participation | How many people refused to participate or dropped out? Reasons? | N/A |
| *Setting* |  |  |
| 14. Setting of data collection | Where was the data collected? e.g. home, clinic, workplace | Methods, p6 |
| 15. Presence of non-participants | Was anyone else present besides the participants and researchers? | N/A |
| 16. Description of sample | What are the important characteristics of the sample? e.g. demographic data, date | Methods, p6, p9; Results p5 |
| *Data collection* |  |  |
| 17. Interview guide | Were questions, prompts, guides provided by the authors? Was it pilot tested? | Methods, p6 |
| 18. Repeat interviews | Were repeat inter views carried out? If yes, how many? | N/A |
| 19. Audio/visual recording | Did the research use audio or visual recording to collect the data? | Methods, p7 |
| 20. Field notes | Were ﬁeld notes made during and/or after the interview or focus group? | Methods, p7 |
| 21. Duration | What was the duration of the interviews or focus group? | Methods, p6 |
| 22. Data saturation | Was data saturation discussed? | Methods, p7 |
| 23. Transcripts returned | Were transcripts returned to participants for comment and/or correction? | N/A |
| **Domain 3: analysis and ﬁndings** |  |  |
| *Data analysis* |  |  |
| 24. Number of data coders | How many data coders coded the data? | Statements and Contributions, p2 |
| 25. Description of the coding tree | Did authors provide a description of the coding tree? | N/A |
| 26. Derivation of themes | Were themes identiﬁed in advance or derived from the data? | Methods, p7 |
| 27. Software | What software, if applicable, was used to manage the data? | N/A |
| 28. Participant checking | Did participants provide feedback on the ﬁndings? | Methods, p7-p9 |
| *Reporting* |  |  |
| 29. Quotations presented | Were participant quotations presented to illustrate the themes/ﬁndings? Was each quotation identiﬁed? e.g. participant number | Results, p6-p22 |
| 30. Data and ﬁndings consistent | Was there consistency between the data presented and the ﬁndings? | Results, p23, Table 4; Discussion p2-5 |
| 31. Clarity of major themes | Were major themes clearly presented in the ﬁndings? | Results, p6-p22 |
| 32. Clarity of minor themes | Is there a description of diverse cases or discussion of minor themes? | N/A |

# **Table 3: Good Reporting of a Mixed Methods Study (GRAMMS) guidelines**

| **GRAMMS Criterion** | **Focus Area** | **Section, paragraph number** |
| --- | --- | --- |
| **1. Justification for mixed methods** | State the justification for utilising mixed methods in this study. | Introduction, p3 |
| **2. Description of design** | Describe the type, priority and sequence of the different methods of study employed. | Methods, p1-6 |
| **3. Methods detail** | Describe the sampling approach for both quantitative and qualitative data collection and description of [data analysis](https://pubrica.com/services/data-analytics-machine-learning/) method for both methods. | Methods, p2, p5 |
| **4. Integration** | State how the quantitative and qualitative data was integrated. | Methods, p6 |
| **5. Method limitations** | Identify the limitations associated with combining the two methods used for the study (mixed methods). | Discussion, p6 |
| **6. Insights from integration** | Provide details of any new knowledge gained through the integration of quantitative and qualitative data. | Discussion, p1-5 |

**Table 4: Research team demographics**

| **Characteristic** | **Categories (n)** |
| --- | --- |
| Gender | Male (2); Female (6) |
| Nationality | Sri Lanka (3); Britain (2); Ireland (2); United States (1) |
| Education | PhD (8) |
| Accreditation | University (8) |
| Expertise | Research methods (8); Assistive technology (5) |

# **Table 5: Odds ratios for functional difficulty and AT indicators, excluding glasses**

|  | **Univariable OR (95% CI)** | **Multivariable OR (95% CI)** |
| --- | --- | --- |
| **Need (excluding glasses)** | | |
| Sex | | |
| Male | 1·00 | 1·00 |
| Female | 1·08 (0·91–1·29) | 1·13 (0·96–1·35) |
| Age | | |
| <18 | 1·00 | 1·00 |
| 18–64 | 2·00‡ (1·53–2·61) | 2·01‡ (1·54–2·62) |
| 65+ | 6·69‡ (4·89–9·14) | 6·72‡ (4·92–9·18) |
| Setting | | |
| Urban | 1·00 | 1·00 |
| Rural | 0·89 (0·69–1·15) | 0·92 (0·71–1·18) |
| Estate | 0·75 (0·45–1·26) | 0·73 (0·43–1·24) |
| **Use (excluding glasses)** | | |
| Sex | | |
| Male | 1·00 | 1·00 |
| Female | 0·72† (0·57–0·91) | 0·76* (0·60–0·97) |
| Age | | |
| <18 | 1·00 | 1·00 |
| 18–64 | 3·50‡  (2·21–5·53) | 3·51‡  (2·22–5·54) |
| 65+ | 9·86‡ (5·94–16·36) | 9·58‡ (5·76–15·93) |
| Setting | | |
| Urban | 1·00 | 1·00 |
| Rural | 0·78 (0·55–1·13) | 0·82 (0·57–1·17) |
| Estate | 0·26† (0·10–0·73) | 0·26† (0·09–0·71) |
| **Unmet need (excluding glasses)** | | |
| Sex | | |
| Male | 1·00 | 1·00 |
| Female | 1·36† (1·11–1·67) | 1·41‡ (1·15–1·73) |
| Age | | |
| <18 | 1·00 | 1·00 |
| 18–64 | 1·53† (1·14–2·07) | 1·54† (1·14–2·09) |
| 65+ | 5·45‡ (3·85–7·70) | 5·55‡ (3·93–7·83) |
| Setting | | |
| Urban | 1·00 | 1·00 |
| Rural | 1·01 (0·76–1·34) | 1·04 (0·78–1·37) |
| Estate | 1·05 (0·59–1·86) | 1·02 (0·57–1·83) |

*Abbreviations: Odds ratio (OR), confidence interval (CI); * p<0·05;* † *p<0·01;* ‡*p<0·001, Logistic regression results are presented odds ratios for univariable (single) and multivariable (joint) models, testing the association of all demographic factors (first individually, then all together) with each AT indicator (need, use, unmet need) and functional difficulty level (any, high), excluding glasses as AT, using weighted survey data.*

# **Table 6**: **Aggregated predicted prevalence for AT indicators, excluding glasses**

|  | | **Need** | **Use** | **Unmet** |
| --- | --- | --- | --- | --- |
|  |  | **Prev % (95% CI)** | **Prev % (95% CI)** | **Prev % (95% CI)** |
| Overall |  | 8·3% (7·5–9·2) | 3·7% (3·1–4·4) | 5·7% (5·1–6·4) |
| Sex |  |  |  |  |
|  | Male | 7·9% (6·9–9·0) | 4·2% (3·4–5·2) | 4·8% (4·1–5·7) |
|  | Female | 8·8% (7·8–9·9) | 3·2% (2·6–3·9) | 6·7% (5·8–7·7) |
| Age |  |  |  |  |
|  | 2–17 | 4·8% (3·8–6·1) | 1·4% (0·9–2·2) | 3·9% (3·0–5·0) |
|  | 18–64 | 9·2% (8·3–10·3) | 4·9% (4·2–5·7) | 5·9% (5·1–6·7) |
|  | 65+ | 25·5% (22·2–29·0) | 12·4% (10·2–15·1) | 18·4% (15·5–21·6) |
| Setting |  |  |  |  |
|  | Urban | 9·1% (7·3–11·2) | 4·6% (3·3–6·5) | 5·6% (4·4–7·1) |
|  | Rural | 8·3% (7·4–9·3) | 3·8% (3·2–4·5) | 5·8% (5·0–6·6) |
|  | Estate | 6·6% (4·3–10·2) | 1·2% (0·5–3·0) | 5·7% (3·5–9·2) |

*Abbreviations: Confidence interval (CI); Predicted prevalence (Prev). Predicted prevalences and 95% CIs are presented for weighted survey data including glasses and aggregated by demographic factors. Prevalence estimates are given with 95% confidence intervals in parentheses.*

# **Figure 1: Disaggregated predicted prevalence for AT indicators, excluding glasses**


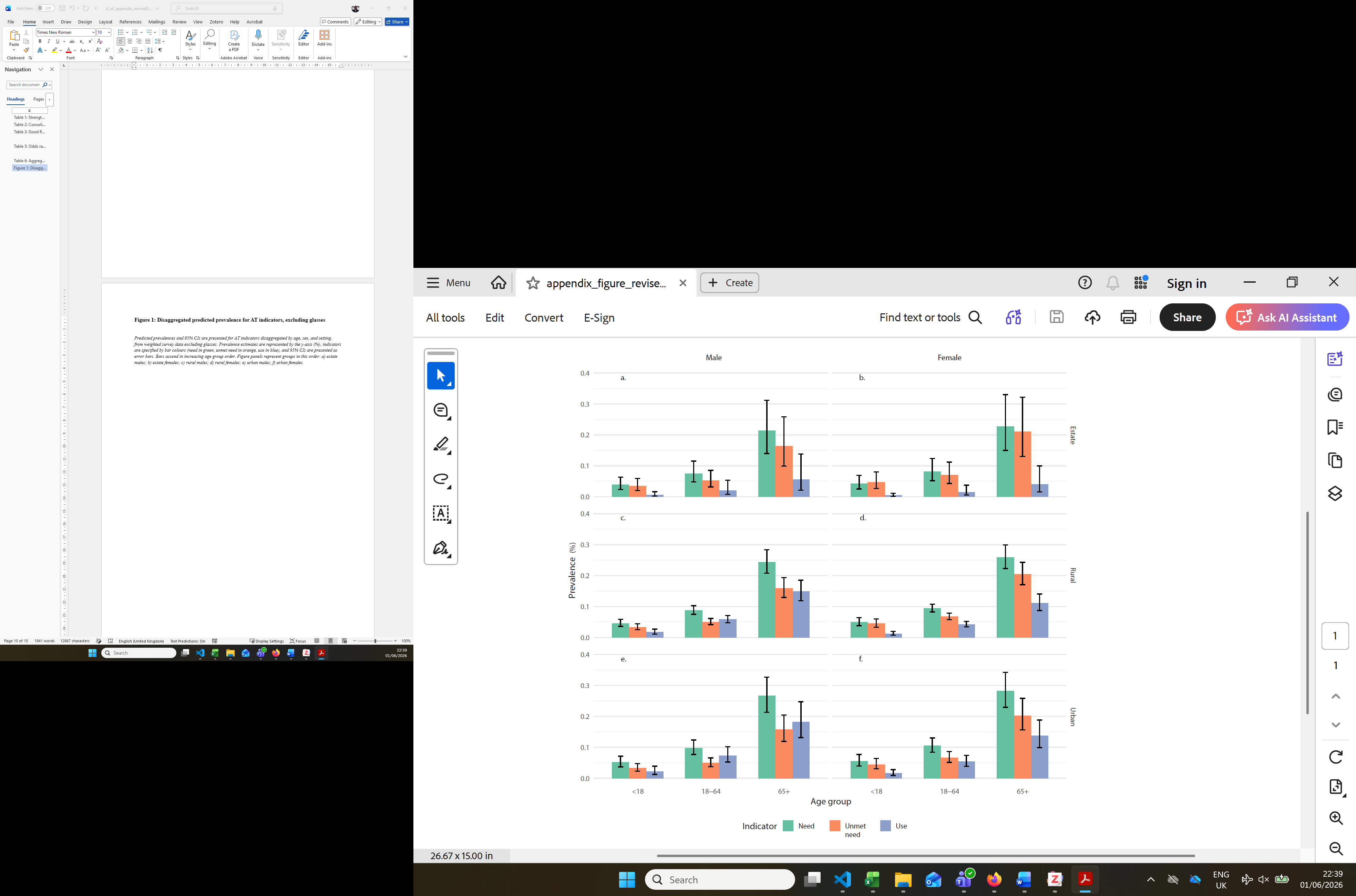


*Predicted prevalences and 95% CIs are presented for AT indicators disaggregated by age, sex, and setting, from weighted survey data excluding glasses. Prevalence estimates are represented by the y-axis (%), indicators are specified by bar colours (need in green, unmet need in orange, use in blue), and 95% CIs are presented as error bars. Bars ascend in increasing age group order. Figure panels represent groups in this order: a) estate males; b) estate females; c) rural males; d) rural females; e) urban males; f) urban females.*
